# Effects of Urbanization on Plant–Pollinator Interactions in the Tropics: An Experimental Approach Using Exotic Plants

**DOI:** 10.3390/insects11110773

**Published:** 2020-11-09

**Authors:** Marie Zakardjian, Benoît Geslin, Valentin Mitran, Evelyne Franquet, Hervé Jourdan

**Affiliations:** 1 IMBE, Aix Marseille Univ, Avignon Université, CNRS, IRD, 13000 Marseille, France; benoit.geslin@imbe.fr (B.G.); valentin.mitran@etu.univ-amu.fr (V.M.); evelyne.franquet@imbe.fr (E.F.); 2 IMBE, Aix Marseille Univ, Avignon Université, CNRS, IRD, Nouméa 98800, New Caledonia; herve.jourdan@imbe.fr

**Keywords:** pollination networks, insular, biological introductions, urban, exotic bees

## Abstract

**Simple Summary:**

Island environments of the Southwest Pacific, like New Caledonia, generally present poorly diversified bee fauna. Thus, they are particularly prone to the establishment of introduced bee species. These exotic species may compete with native bees for plant resources, disrupt pollination of native plants, and enhance the reproduction of exotic ones. To conserve local plant–pollinator interactions, it is essential to assess the factors favoring the presence and the activity of exotic bees. Here, we focused on the effects of urbanization on plant–pollinator interactions. We set up experimental plant communities composed of four exotic species in two contrasted habitats—a natural environment vs. an urban environment—and observed plant–pollinator interactions. We showed that the urban environment was largely dominated by exotic bees. We also showed that some exotic bee species can interact preferentially with a single exotic ornamental plant species. Overall, our results indicate that Nouméa is an entry point for exotic bees, which should encourage local authorities to maintain biosecurity measures to effectively limit the arrival of exogenous bees. Lastly, the use of exotic horticultural plants in green public spaces should be questioned regarding their potential attractiveness to exotic bees.

**Abstract:**

Land-use changes through urbanization and biological invasions both threaten plant-pollinator networks. Urban areas host modified bee communities and are characterized by high proportions of exotic plants. Exotic species, either animals or plants, may compete with native species and disrupt plant–pollinator interactions. These threats are heightened in insular systems of the Southwest Pacific, where the bee fauna is generally poor and ecological networks are simplified. However, the impacts of these factors have seldom been studied in tropical contexts. To explore those questions, we installed experimental exotic plant communities in urban and natural contexts in New Caledonia, a plant diversity hotspot. For four weeks, we observed plant–pollinator interactions between local pollinators and our experimental exotic plant communities. We found a significantly higher foraging activity of exotic wild bees within the city, together with a strong plant–pollinator association between two exotic species. However, contrary to our expectations, the landscape context (urban vs. natural) had no effect on the activity of native bees. These results raise issues concerning how species introduced in plant–pollinator networks will impact the reproductive success of both native and exotic plants. Furthermore, the urban system could act as a springboard for alien species to disperse in natural systems and even invade them, leading to conservation concerns.

## 1. Introduction

Land-use changes, including urbanization, are the major cause of pollinator decline on a global scale [1,2]. However, there is no clear consensus about the established negative effects of urban areas on pollinator communities [3,4]. Depending on the study, cities can be perceived as either sinks or refuges for bees [4,5,6,7]. For example, bee diversity and/or abundance have been observed to decline along urbanization gradients [3,5,6,8,9]. Yet cities have the capacity to maintain numerous bee species because of high habitat heterogeneity, together with abundant floral resources available throughout the year [4,7,10,11]. Some cities even host more bee species compared to adjacent agricultural areas (e.g., [4,12,13,14]).

Several studies recommend fostering native plants to promote bees in cities (e.g., [15]). Indeed, a higher proportion of native species is supposed to increase bee activity [16] and abundance, notably for native bees ([10,17,18,19,20], but see [21]). However, urban flora is largely composed of horticultural and often exotic plants [22,23]. Those species, by extending the flowering season [24] and offering a more abundant floral resource, may have positive effects on pollinators. For example, Matteson and Langellotto [25] found that some megachilid bees were more active on exotic plants in urban community gardens. Thus, by increasing the abundance and activity of pollinators, exotic plants may facilitate the pollination of native ones [26,27]. However, the presence of exotic plants could also have negative effects on native fauna and flora. Hence, some studies have described competition between exotic plants and native ones [27]. For example, in a natural area, Kaiser-Bunbury et al. [28] showed higher fruit production for native plants in sites where exotic plants were removed. Thus, exotic plants may compete with native plants by attracting all or part of their pollinators.

Regarding pollinators, the presence of exotic plants in urban areas may sustain exotic pollinators [29,30]. However, those species could have important impacts on native bee communities by disrupting native plant–pollinator networks. First, exotic bees may compete with native bees for floral resources [31] and/or nesting sites [32], potentially driving a decrease in the abundance and the activity of native bees. Regarding plant species, because they did not coevolve together, exotic bees may not be efficient pollinators of native plants due to morphological mismatches. Moreover, exotic bees tend to be more active on exotic plants than on native ones [33], potentially leading to a promotion of exotic plants at the expense of native ones [31]. Such a synergistic association, whereby exotic invasive species promote each other, is referred to as “invasional meltdown” [34].

These threats are amplified in insular systems, which generally present simplified plant–pollinator networks [35]. These systems, harboring depauperate bee fauna, as in the Southwest Pacific (SWP) archipelagos [36], offer a great opportunity for exotic bees to integrate into plant–pollinator networks due to vacant ecological niches [37,38,39,40,41,42]. However, even though exotic bees may have several negative impacts on native communities, recent results showing the super-generalist foraging behavior of a native bee in a SWP archipelago [43] challenged previous concerns about exploitative competition between native and exotic bees.

To our knowledge, the effects of urbanization on plant–pollinator interactions have never been explored in tropical islands. In this study, we set up experimental exotic plant communities in both urban and natural contexts in New Caledonia (SWP) and surveyed their interactions with local bee fauna, either exotic or native. The New Caledonian bee community is composed of merely 38 native species and, until now, counted five exotic bee species [44,45,46,47,48]. We assessed (i) the activity (i.e., the number of interactions realized) of exotic and native bees on experimental exotic plant communities in urban vs. natural contexts and (ii) the overall visitations received by experimental exotic plant communities in urban vs. natural contexts. We hypothesize that tropical urban areas promote exotic plant–pollinator interactions, resulting in the following:

A higher number of visits realized by exotic bees in urban sites than in natural sites, with the opposite effect on native bees.

A higher number of visits received by experimental exotic plant communities in urban sites than in natural sites.

## 2. Materials and Methods

### 2.1. Study Area and Site Selection

This study was conducted in New Caledonia on the Grande Terre Island. Located in the intertropical region of the SWP (−22.27631 166.4572), average annual temperatures and precipitations on the archipelago are 23.5 °C and 1070 mm (average values for 1981–2010; [49]). Regarded as the second greatest plant biodiversity hotspot on the global scale (in terms of endemic plants per unit area; [50]), New Caledonia counts more than 3000 angiosperm species, of which 77.7% are endemics [51]. In particular, the island presents a vegetation type called “ultramafic shrubland”, growing on an ultramafic substrate and hosting a very singular flora composed of 96.9% of endemic species [50]. Despite a remarkable plant diversity, New Caledonia hosts only 38 native bee species. This limited diversity of wild bees could be partly explained by the geological isolation of New Caledonia, which occurred approximately during the emergence of the Apoidea family [44].

This study was conducted at six sites: three in an urban context (i.e., Nouméa) and three in a natural context (i.e., Tontouta valley). Nouméa is the main city of the archipelago, with 99,926 inhabitants for an area of 45.7 km² [52]. Within the city, the three sites sampled, separated by at least 3 km, were (i) the park of the French National Research Institute for Sustainable Development (Institut de Recherche pour le Développement, IRD), with six registered beehives, (ii) the Parc Zoologique et Forestier Michel Corbasson (PZF), with two registered beehives, and (iii) the Tjibaou Cultural Centre (TJI), with three registered beehives. Tontouta valley, the natural area, is located 30 km north of Nouméa. The plant formation of the valley is an ultramafic shrubland subject to frequent fires [50], with a high proportion of endemic plants [53]. In contrast to the urban area, there is no registered beehive within the valley. However, Tontouta valley is disrupted by mining activities. For accessibility reasons, the sites (i.e., “TTA1”, “TTA2” and “TTA3”) were placed close to the path leading to the mine. Finally, sites were separated by at least 3 km in order to match the urban conditions.

### 2.2. Experimental Exotic Plant Communities

**Figure 1:**
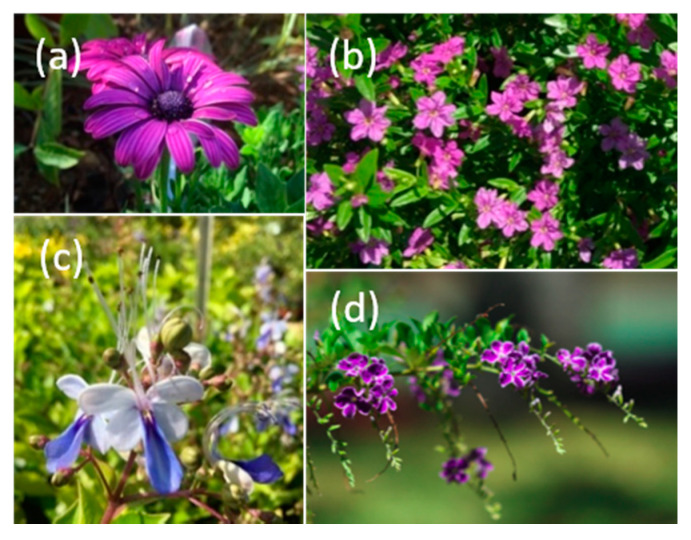
Species of the experimental exotic plant communities: (**a**) *Osteospermum* sp., (**b**) *Cuphea hyssopifolia*, (**c**) *Clerodendrum ugandense*, and (**d**) *Duranta erecta*.

Experimental exotic plant communities (Figure 1) were placed in each site. These communities were composed of four exotic species, with four individual plants per species: *Duranta erecta* L., 1753 (Verbenaceae); *Clerodendrum ugandense* Prain, 1909 (*syn. Rotheca myricoides* (Hochst.) Steane & Mabb., 1998; Lamiaceae); *Osteospermum* sp. (Asteraceae); *Cuphea hyssopifolia* Kunth, 1823 (Lythraceae). These species were selected so that they (i) produced flowers during the whole sampling season, (ii) had similar flower colors based on human perception to limit potential bias in the attractivity of flowers to pollinators (even if a measure of the spectral reflectance with a spectrometer should have been better), and (iii) were available in large quantities in nurseries (Botanea Pépinière, Dumbea, New Caledonia). That is why the experimental plant communities were only composed of exotic species: there is a lack of native plants in commercial nurseries.

### 2.3. Sampling

**Figure A1:**
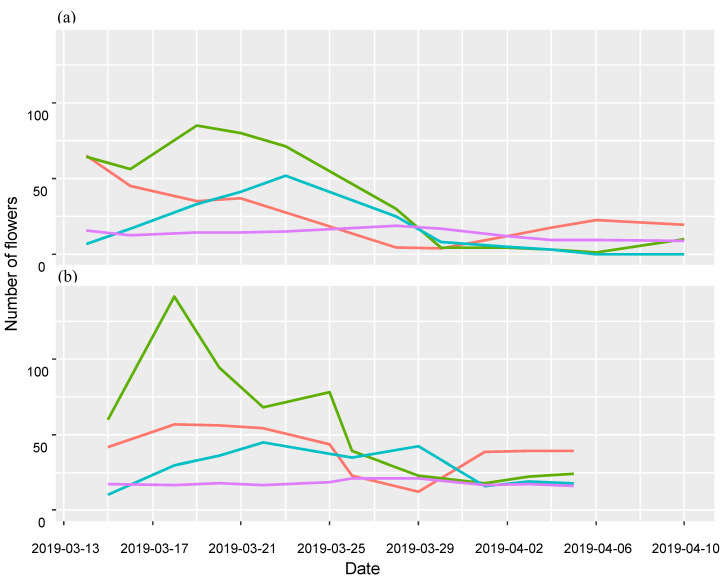
Number of flowers of each species of the experimental exotic plant communities at each day of observation in (**a**) the urban context and (**b**) the natural context. For *Duranta erecta* (pink) and *Cuphea hyssopifolia* (purple), the actual number of flowers was divided by ten to fit the scale of *Clerodendrum ugandense* (green) and *Osteospermum* sp. (blue).

The experimental exotic plant communities were installed on 13 March 2019 when the four species were blooming simultaneously, and plant–pollinator interactions were observed from 14 March 2019 to 10 April 2019. However, due to the end of the flowering period of the experimental exotic plant communities, observations realized after 6 April 2019 were not included in the statistical analysis (Figure A1). Thus, in each experimental exotic plant community, plant–pollinator interactions were effectively observed 10 times from 14 March 2019 to 6 April 2019. Observations were made during non-rainy and no- or low-wind days. During these days, observations were made between 8:00 a.m. and 12:00 p.m. (i.e., before the hottest hours of the day) to avoid bias linked to a decrease in pollinators’ activity. Each observation consisted of rounds of 10 min, where all plant-pollinator interactions were recorded. We counted an interaction/visit when a pollinator contacted the reproductive organs of a plant. The identity of the pollinator and the number of flowers visited were recorded. Finally, for each observation, the total number of open flowers were counted for each plant species.

Preliminary identifications were made during field observations. The low diversity of bees in New Caledonia allowed us to visually identify most pollinators to the species level during the observations. Moreover, due to their large body size, most bees observed during this study were easily identified while foraging on flowers or while flying between flowers. *Amegilla pulchra*, *Apis mellifera*, and *Megachile laticeps* have strong morphological differences enforcing the degree of confidence that can be assigned to the identifications. Concerning small-body-sized bees, only two genera were encountered during field sampling, namely, *Homalictus* spp. and *Braunsapis* spp. Species of the latter present a white facial mask, which made it easy to discriminate between these two genera during field observations.

Nevertheless, 10 min of captures were performed after each 10 min of observations to bring back specimens to the laboratory to verify identifications. To identify bees to the species level, we used Pauly et al. [48] for *Homalictus* spp., Pauly and Munzinger [44] for *Megachile* spp., Pauly et al. [47] for *Austronomia* spp., and Leijs et al. [54] for *Amegilla* spp. Due to the absence of relevant identification keys, *Braunsapis* spp. were only identified to the genus level.

### 2.4. Data Analysis

Statistical analyses were performed using R 3.6.1. [55] and Rstudio 1.2.1335 [56].

To assess the impact of urbanization on the activity of bees, the response variables were the number of interactions realized, respectively, by (i) exotic bees except honeybees (i.e., exotic wild bees), (ii) honeybees, and (iii) native bees on the experimental exotic plants. Honeybees were analyzed separately due to their sociality. Indeed, a honeybee colony can reach 20,000 to 60,000 workers, which is a hundredfold the abundance of a solitary bee population [31]. Thus, their numerical dominance could have either induced a significant response at the level of the community of exotic bees or buffered a significant response from the solitary bees. The explanatory variables tested were the context (urban vs. natural), sites and the number of flowers of the experimental exotic plant communities. Then, to assess the impact of urbanization on the number of visits received by exotic plants, the response variables tested were the number of visits received by (i) the experimental exotic plant communities and (ii) each of the species composing the communities. In the first case, the explanatory variables tested were the context (urban vs. natural), sites, and the number of flowers of the experimental exotic plant communities. In the second case, the explanatory variables tested were the context (urban vs. natural), sites, the number of flowers of the species tested, and the number of flowers of the other species of the experimental exotic plant communities.

Due to different numbers of zeros depending on the response variables (from 32% to 90%), we systematically tested four types of statistical models and checked for over- or subdispersion. The two first models were generalized linear mixed-effects models (GLMMs) with (i) Poisson error distribution and (ii) negative binomial error distribution. Sites were added as a random effect to account for pseudoreplication. The two other models were zero-inflated models (ZIMs) with (i) Poisson distance and (ii) negative binomial distance. Models with dispersion scores closest to one were selected. Thus, GLMMs with negative binomial error distribution were used for the number of visits received, respectively, by *D. erecta* and *Osteospermum* sp., a ZIM with Poisson distance was used for the number of visits received by *C. hyssopifolia*, and ZIMs with negative binomial distance were used for the number of interactions realized, respectively, by honeybees and native bees and the number of visits received, respectively, by the experimental exotic plant communities and *C. ugandense*.

### 2.5. Plant–Pollinator Networks

Plant–pollinator networks were obtained using the package Bipartite [57], implemented in R 3.6.1. [55] and Rstudio 1.2.1335 [56].

To build the plant–pollinator networks, data were transformed and then pooled to obtain one network for the urban context and one network for the natural context. First, data were transformed according to the following equation:

$$\frac{x_{i}\;\times \;n_{i}}{1000},$$

with $x_{i}$ being the number of interactions realized by a bee species on a plant species in a site during one session and $n_{i}$ being the number of flowers of this plant species. Then, these values were averaged for each landscape context (i.e., urban and natural) to obtain the networks.

Two network topology indices were calculated for each network. First, the specialization index (i.e., d’) was calculated. This index measures the deviation of a bee species from random associations with the plant species in the network and ranges from zero (no specialization) to one (perfect specialization). Then, Müller’s index was calculated, following the formula given by Müller et al. [58]. This index, also referred to as the Potential for Apparent Competition, measures how much a bee species (i.e., the “acting bee”) contributes to the visits received by the plant species it shares with another bee species (i.e., the “target bee”; see [59]). Müller’s index ranges from zero (no plant species shared) to one (all visits realized on the same plant species, and, thus, there is a high potential for the acting bee to influence the target bee via shared plant species).

## 3. Results

We recorded 458 interactions during the experiment: 313 in the urban context and 145 in the natural context.

In the urban context, we observed four to five exotic bee species belonging to four genera: *Apis mellifera*, *Amegilla pulchra*, *Megachile laticeps*, and one or two *Braunsapis* spp. (two morphospecies were captured, and their identification remains to be confirmed through DNA barcoding). The only native bee species observed in the urban context were *Homalictus* spp.: *Homalictus aponi* and *Homalictus risbeci*.

In the natural context, the only exotic bee species observed was *A. mellifera*. We observed four native species belonging to three genera: *H. aponi*, *H. risbeci*, *Austronomia sichelli*, and *Megachile albomarginata*.

### 3.1. Bee Visits in Relation with the Landscape Context

- The effect of urbanization on the number of visits realized by exotic wild bees could not be tested statistically simply because exotic wild bees realized no interaction in the natural context. In contrast, exotic wild bees realized 185 interactions in the urban context.
- Regarding honeybees, the landscape context had no effect on their number of interactions realized. Only the number of flowers of the experimental exotic plant communities had a significant positive effect on the number of interactions realized by honeybees (estimate = 2.60; *p* = 3.86 × 10^−7^ ***).
- None of the variables tested had a significant effect on the number of interactions realized by native bees.

### 3.2. Bee Visits within the Experimental Exotic Plant Communities

**Figure 2:**
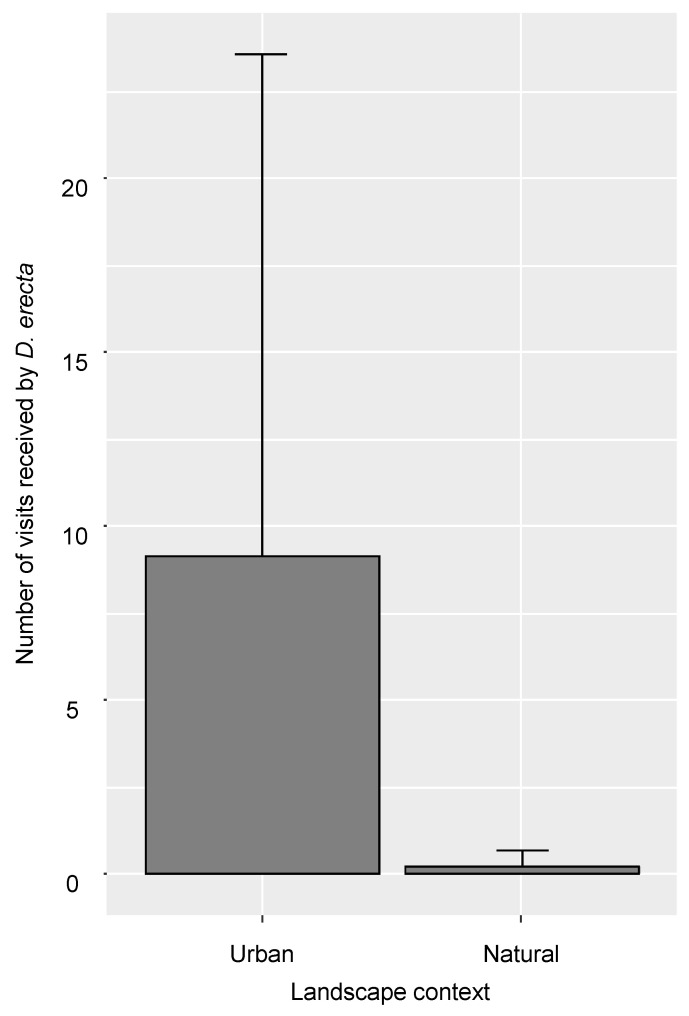
Mean (±SD) number of visits received by *D. erecta* in the urban context and the natural context.

- Neither the landscape context nor the number of flowers of the experimental exotic plant communities had a significant effect on the number of visits received by the experimental exotic plant communities. However, the number of visits received by the experimental exotic plant communities was significantly lower in sites TTA2 (estimate = −1.70; *p* = 8.154 × 10^−3^ **) and TTA3 (estimate = −2.45; *p* = 8.57 × 10^−4^ ***), both in the natural area.
- Regarding the experimental exotic plant communities at the species level, the number of visits received by *D. erecta* was significantly higher in the urban context (estimate = 3.70; *p* = 3.75 × 10^−7^ ***; Figure 2). The number of visits received by *D. erecta* also significantly increased with its number of flowers (estimate = 0.89; *p* = 1.22 × 10^−2^ *). The landscape context had no effect on the number of visits received by *Osteospermum* sp. However, the number of visits received by *Osteospermum* sp. significantly increased with its number of flowers (estimate = 1.57; *p* = 2.49 × 10^−4^ ***) and significantly decreased with the number of flowers of the other species of the experimental exotic plant communities (estimate = −1.63; *p* = 1.2006 × 10^−2^ *). Finally, none of the variables tested influenced the number of visits received by either *C. ugandense* or *C. hyssopifolia*.

### 3.3. Plant–Pollinator Networks

**Figure 3:**
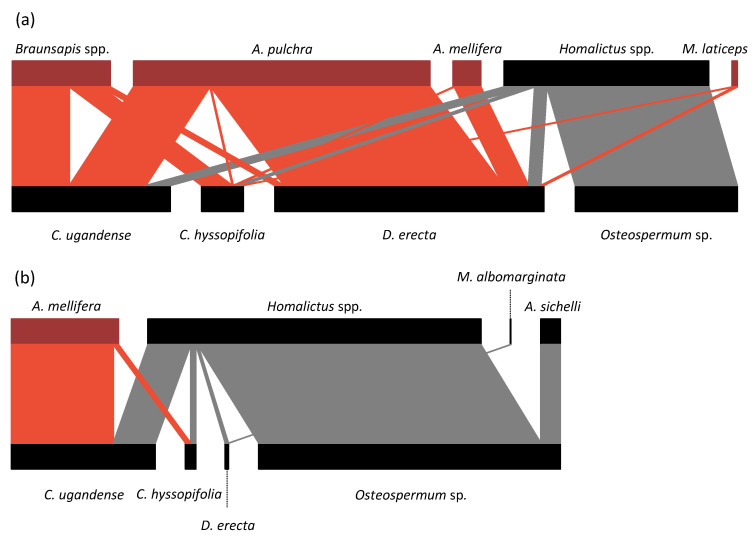
Plant–pollinator networks for (**a**) the urban context and (**b**) the natural context. Higher trophic levels represent bee taxa, and lower trophic levels represent species of the exotic experimental plant communities. The width of the links is proportional to the average number of interactions realized per 1000 flowers between two species. Exotic bee taxa and their interactions are illustrated in red.

**Figure 4:**
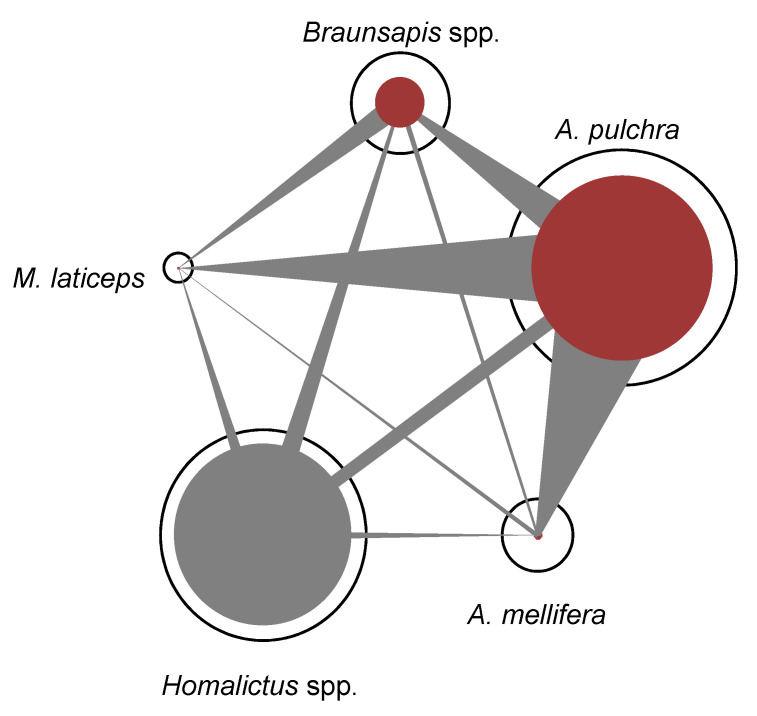
Competition network for the urban context plotted from Müller’s index values. Black circles are proportional to the number of interactions realized by each bee taxon. Solid circles are proportional to the potential for apparent competition at the species level undergone by each bee taxon. Bars connecting the taxa are proportional to the potential for apparent competition exerted by the acting bee taxa on the target bee taxa. Exotic bee taxa are illustrated in red.

- Regarding the specialization index, in the urban context, the most specialized bee species was *A. pulchra* (d’ = 0.68), followed by *Homalictus* spp. (d’ = 0.63), *Braunsapis* spp. (d’ = 0.52), *A. mellifera* (d’ = 0.25), and *M. laticeps* (d’ = 0.13). *Amegilla pulchra* had the highest specialization index because it realized almost all of its interactions on *D. erecta*, making it the most visited plant species of the urban plant–pollinator network (Figure 3). In the natural context, the most specialized bee species was *M. albomarginata* (d’ = 0.70), followed by *A. mellifera* (d’ = 0.68), *Homalictus* spp. (d’ = 0.30), and *A. sichelli* (d’ = 0.12).
- Regarding Müller’s index, in the urban context (Figure 4), *A. pulchra* had the highest values as an acting bee compared to the other bee species (Table 1). *Homalictus* spp. had the highest intraspecific Müller’s index value (Table 1). In the natural context, *Homalitcus* spp. had both the highest Müller’s index values as an acting bee compared to the other bee species and the highest intraspecific Müller’s index value (Table 1).

## 4. Discussion

In the present study, we investigated, on a tropical island, the impact of the landscape context (urban vs. natural) on bee communities and their interactions with experimental plant communities composed of four exotic species. We observed a high contrast between the two landscape contexts: we did not observe any exotic wild bees in the natural context, while they dominated the plant–pollinator network in the urban context. Conversely, the landscape context had no effect on the number of visits realized by both *A. mellifera* and native bees. Regarding the experimental exotic plant communities, the urban context had no effect at the community level. However, at the species level, the exotic species *D. erecta* was more visited in the urban context. Regarding the network topology indices, the most specialized bee species, according to the specialization index, was *A. pulchra* because this species realized almost all of its interactions on *D. erecta*, making it the most visited plant species of the urban plant–pollinator network. Furthermore, *Homalictus* spp. were more specialized in the urban context than in the natural context. Finally, *A. pulchra* had the highest Müller’s index values compared to any other bee species in the urban context, and *Homalictus* spp. showed the highest Müller’s index values in the natural context.

### 4.1. Exotic Wild Bees

Visits of exotic wild bees (i.e., *A. pulchra*, *Braunsapis* spp., and *M. laticeps*) on experimental exotic plant communities were only observed in the urban context, while no individuals were observed in the natural context. This underlines that the urban and natural contexts hosted two contrasting bee communities. Exotic wild bees were only present in the urban context, where they dominated the network (Figure 3). First, this is likely related to the inherent conditions of the urban context. Cities are entry points for exotic species due to human activities and trade flows [60], and Nouméa is no exception with its commercial harbor. Furthermore, as in Nouméa, many other cities worldwide host numerous exotic plant species [22], which can potentially promote the presence of exotic bees [29]. This result is consistent with other studies conducted in temperate areas (e.g., [61,62]) and shows that insular tropical cities may also promote the presence of exotic wild bees. Second, the absence of exotic wild bees in the natural context is probably due to the conditions of the Tontouta valley. Indeed, this valley is located on an ultramafic substrate, with high concentrations of heavy metals and low levels of nutrients. Thus, this soil may represent an abiotic barrier to the establishment of exotic plants that are not adapted to it. Thus, ultramafic shrublands could be relatively preserved from the spread of exotic wild bees. Conversely, exotic wild bees may disperse preferentially along the coastline, which is constituted of volcano-sedimentary soils that are more favorable to the establishment of exotic plants. We are currently developing a project to understand how exotic wild bees will further disperse within the New Caledonian territory.

### 4.2. Case of the Human Managed Honeybee

The only exotic bee observed in the natural context was the human-managed species *A. mellifera*. This eusocial species is native to Eurasia and was imported to New Caledonia in 1848 for beekeeping activities [63]. *Apis mellifera* was as active in the natural context than in the urban context. Its capacity to colonize the natural context and to become established in the ultramafic shrubland is, therefore, mainly due to human management and the establishment of apiaries. Although no beekeepers were officially reported by local authorities in the Tontouta valley, we found an apiary of a few hives during the field season. *Apis mellifera* is a super-generalist species that can forage on many wild plant species. This may facilitate its persistence in the natural context and encourage local beekeepers to produce honey from native plant species. This raises serious concerns about its potential impact on wild fauna and flora and, therefore, on the sustainability of native communities in natural areas. Indeed, social bees were originally absent in New Caledonia, such as in many SWP islands, and introducing populous colonies of *A. mellifera* in an environment is never a zero-sum game. Its impacts on native fauna and flora have been largely studied and reviewed in [31,64]. Compared to native bees, honeybees are numerically dominants due to their eusocial nature, they visit more flowers at a broader spatial scale and are active for most of the year [65,66,67]. Thus, they can outcompete wild solitary bees, leading to a decrease in the activity and abundance of wild bee species, potentially and ultimately leading to local extinctions. This trend could also occur in cities. Indeed, a recent study showed that a high density of beehives had a negative impact on the foraging activity of wild bees in an urban context [23]. Additionally, honeybees can decrease the reproductive success of local plants through overvisitation [68] or inefficient pollination [69,70].

### 4.3. Recent Introductions of Exotic Bees

In the present study, we detected, for the first time, one to three new exotic bees in the New Caledonian territory: probably a second and yet unidentified *Amegilla* sp. and one or two yet unidentified *Braunsapis* spp., a genus hitherto absent from New Caledonia. Genetical analyses are currently ongoing to confirm the identity of the specimen of *Amegilla* sp. and whether the two types of facial masks, observed among the *Braunsapis* spp. captured, reveal the existence of two different species or are related to intraspecific phenotypic variation. These recent discoveries are in line with the global trend that the rate of first introductions of exotic species does not show any sign of saturation [71]. Instead, this rate is increasing for insects, and the number of exotic bees introduced out of their natural range has widely risen worldwide in the past decades [32]. As an example, *Braunsapis puangensis* has been recorded in Fiji in 2007 and is now widespread in this archipelago and also present in French Polynesia [72,73]. Since New Caledonia is on the path of invasion followed by *B. puagensis* [72], it is possible that one of the unidentified *Braunsapis* spp. recorded in the present study is *B. puangensis*. This remains to be confirmed through DNA barcoding using mitochondrial gene cytochrome oxidase I [74]. Furthermore, *A. pulchra* is another apid bee that was recently introduced in the SWP. This species was first detected in New Caledonia in 2016 and has rapidly spread within the territory since then. *Amegilla pulchra* has also spread in Fiji and French Polynesia [72,73]. Finally, we may have recorded a new *Amegilla* sp. that has not been previously detected, supporting the fact that some apid bee species are currently invading the SWP [72,73]. All those species are long-tongued bees, unlike the majority of SWP native species. Thus, they could potentially disrupt native plant–pollinator interactions by foraging a broader range of plants and favoring their reproduction and dissemination. Indeed, some studies have demonstrated that exotic long-tongued bees could be able to outcompete native bees such as *Homalictus* spp. and promote the spread of exotic plants that are undervisited by native bees [72,73,75].

### 4.4. Native Bees

In this study, the urban context had no effect on the activity of native bees. We expected a decrease in the activity of native bees in the urban context due to a reduced abundance of native plants and an increased activity of exotic bees. Instead, we found that native bees realized as many interactions in the natural context as in the urban context. However, all those interactions were realized by a single genus (*Homalictus* spp.), and we did not detect any other native genera in the city. This unexpected result may be due to the generalist foraging behavior of some native bees in several insular systems. A study conducted in Azores [39] stated that native bees could be super-generalist in insular systems where bee fauna is depauperate. In those systems, intraspecific competition could be greater than interspecific competition, selecting for generalist foraging behavior instead of specialization. In Fiji, a neighboring archipelago to New Caledonia, the most common native bees belong to the genus *Homalictus* and represent supergeneralists characterized by a broader diet than honeybees [43]. *Homalitcus* is also the most common native genus in New Caledonia and the only one observed in the urban context. Furthermore, *Homalictus* spp. showed the highest intraspecific Müller’s index values and low interspecific Müller’s index values in both the natural and urban contexts. These results show that the intraspecific competition might prevail for *Homalictus* spp. (Figure 4), as expected, according to the supergeneralist hypothesis [39]. Thus, if *Homalictus* spp. have supergeneralist foraging behavior in New Caledonia too, they could (i) forage indiscriminately on native and exotic plants and (ii) be able to avoid competitive exclusion induced by exotic bees by switching to less-exploited plants. Here, *Homalictus* spp. had a higher specialization index in the urban context than in the natural context. Therefore, we hypothesize that *Homalictus* spp. may have narrowed their diet in the city in response to the competition induced by exotic bees.

### 4.5. Experimental Exotic Plant Communities: A Potential Invasional Meltdown?

The landscape context had no effect on the total number of visits received by experimental exotic plant communities. Globally, experimental exotic plants were visited as much in the urban context as in the natural context, showing that exotic plants attract both native and exotic bee species. Exotic plants may compete with native ones by attracting all or part of their pollinators, and this could lead to a decrease in native plants’ reproductive success.

At the species level, *D. erecta* was more frequently visited in the urban context, mainly due to the presence of *A. pulchra* (Figure 3). This result is consistent with observations realized in Fiji, where *A. pulchra* also maintains preferential interactions with exotic plant species [76]. *Duranta erecta* is easy to find in New Caledonian horticulturists’ shops and is widely used by Noumeans as an ornamental plant. Under certain conditions, authors have suggested that the diversity of plant species used in gardens could be supplemented by the addition of some exotic species in order to extend the flowering period and produce resources for certain specialist groups of pollinators (e.g., [20]). However, in most cases, it seems that native plants are more important for the sustainability of native bee communities in cities [4,10,16]. In our case, *D. erecta* seems to be the main resource of a single wild species that is itself exotic—*A. pulchra*, which had the highest specialization index in the urban context. Without the presence of *A. pulchra*, *D. erecta* received extremely few visits in the natural context (Figure 3). A positive and synergistic interaction between two invasive species is called an invasional meltdown [34]. Here, *A. pulchra* and *D. erecta* are not yet considered invasive species; therefore, it is too early to characterize the interaction we observed as an invasional meltdown. However, these two species form a module within the urban plant–pollinator network and could promote the spread of each other, which might lead to an invasional meltdown. According to Müller’s index in the urban context, *A. pulchra* is the most potential apparent competitor for every other bee species of the plant–pollinator network. This result suggests that *A. pulchra* has the potential to dominate the network in the presence of *D. erecta*. Further studies are needed to determine whether this bee species can increase the reproductive success of the exotic species *D. erecta*, accelerating its spread.

## 5. Conclusions

Most studies investigating the effects of urbanization on pollinators have been realized in temperate regions. The present study is one of the first conducted in a tropical region and showed that the urban and natural contexts presented highly contrasted bee communities, with a strong urban context/exotic bees’ couple. Thus, Nouméa seems to be an entry point for exotic bees and might act as a springboard for these bees to spread toward other environments. As there were no exotic wild bees in the ultramafic shrubland, we think that exotic bees will mainly spread into natural or seminatural systems on nonultramafic soils (e.g., along the coastline). Unexpectedly, the activity of native bees was not affected by the presence of exotic bees, even if this activity was driven by a single genus. Native bees in insular systems may be generalists and indiscriminately use native and exotic plants.

The first main limitation of the present study is that the experimental design did not include native plants. This was due to a lack of native species in commercial nurseries in New Caledonia. This shows that the supply of ornamental plants is biased in favor of exotic species. Given the importance of native plants as food resources for native bee species, we recommend promoting the production of native plants in nurseries and their use by citizens and entities responsible for managing green spaces. The other main limitation of our study is that we did not account for the plant species present in the sites besides the experimental exotic plant communities, which can modulate the relative attractiveness of our experimental communities in the different contexts. Indeed, plant–pollinator networks are shaped by both competitive interactions between pollinators and the surrounding floral resources. Thus, we were not able to fully explain some differences observed between the two contexts (e.g., why *A. mellifera* was visiting more *D. erecta* in the urban context but more *C. ugandense* in the natural context).

Further studies involving native plants are needed to determine whether exotic plants will take over all or part of the pollinators of native plants, potentially reducing their reproductive success. Furthermore, it would be necessary to monitor the reproductive success of exotic plants involved in strong exotic mutualisms to assess the pollination efficiency of exotic bees.

## Figures and Tables

**Table 1:** Müller’s index calculated from the plant–pollinator networks for (**a**) the urban context and (**b**) the natural context. Columns and rows correspond to bees exerting potential apparent competition (i.e., acting bees) and bees being the target of potential apparent competition (i.e., target bees), respectively. Therefore the diagonals show apparent intraspecific competition.

**(a)**					
	***Braunsapis*** **spp.**	***A.*** ***pulchra***	***A. mellifera***	***Homalictus*** **spp.**	***M. laticeps***
*Braunsapis* spp.	0.503	0.293	0.032	0.143	0.029
*A. pulchra*	0.054	0.787	0.075	0.076	0.008
*A. mellifera*	0.061	0.767	0.104	0.055	0.013
*Homalictus* spp.	0.033	0.095	0.007	0.863	0.002
*M. laticeps*	0.314	0.489	0.078	0.095	0.024
**(b)**					
	***A. mellifera***	***Homalictus*** **spp.**	***M. albomarginata***	***A. sichelli***	
*A. mellifera*	0.695	0.305	0	0	
*Homalictus* spp.	0.098	0.844	0.001	0.056	
*M. albomarginata*	0	0.856	0.144	0	
*A. sichelli*	0	0.933	0	0.066
